# Supplementary material for: BPP43_05035 is a Brachyspira pilosicoli cell surface adhesin that weakens the integrity of the epithelial barrier during infection
Source: Gut Microbes. 2024 Sep 30;16(1):2409247. doi: 10.1080/19490976.2024.2409247 (PMC11444514; doi:10.1080/19490976.2024.2409247)
Supplement: Supplemental material_rev1.docx [file KGMI_A_2409247_SM4488.docx]

**Supplemental material**

**BPP43_05035 is a *Brachyspira pilosicoli* cell surface adhesin that weakens the integrity of the epithelial barrier during infection**

Anandi Rajan^1^, Pablo Gallego^1^, Brendan Dolan^1^, Piyush Patel^2^, Chinmay Dwibedi^2^, Ana S. Luis^1^, Sergio Trillo-Muyo^1^, Liisa Arike^1^, Sjoerd van der Post^1^, Magnus Simrén^2,3^, and Thaher Pelaseyed^1^*

1. Department of Medical Biochemistry and Cell Biology, Institute of Biomedicine, University of Gothenburg, Gothenburg, Sweden.

2. Department of Molecular and Clinical Medicine, Institute of Medicine, University of Gothenburg, Gothenburg, Sweden

3. Center for Functional GI and Motility Disorders, University of North Carolina, Chapel Hill, North Carolina.

*Corresponding author. Email thaher.pelaseyed@medkem.gu.se

**Figure S1. Sequence and structural homologs of BPP43_05035 identified by DALI.**

**(A)** Ten top hits generated by BLAST search of the BPP43_05035 amino acid sequence.

**(B)** Eight top hits generated by DALI search of the BPP43_05035 crystal structure.

**(C)** Structural alignment and projections of BPP43_05035 (7ZAO) (brown), NanA (2AY7) (blue), and NanI (2BF6) (green).

**Figure S2. Hydrolysis assay using 4-nitrophenyl esters as substrates at pH 7.**

**(A)** A cartoon representation of the putative catalytic triad in BPP43_05035. Histidine (H) 221, glutamate (E) 282, and serine (S) 340 are depicted.

**(B)** Activity of BPP43_05035 (10 μM) against 0.5% of PGM type II and III across pH 5.5-8.5. Arrowheads point to sialic acid used as a standard (Std).

**(C)** A cartoon representation showing the overlay of *Vibrio cholerae* sialidase NanH (PDB ID 1W0P) (blue) and BPP43_05035 (PDB ID 7ZAO) (brown). Residues in the catalytic site of NanH are overlayed with the residues in the putative catalytic site of BPP43_05035.

**(D)** Activity of BPP43_05035 (0-10 μM) against 1 mM 4-nitrophenyl acetate across pH 5.5-8.5. n=2 for each group. Data are means ± SD. Significance at each pH was determined by unpaired t-test.

**(E)** Activity of BPP43_05035 (10 μM) against (10 nM-1 µM) 4-nitrophenol, 4-nitrophenyl butyrate, and 4-nitrophenyl octanoate at pH 7.0.
